# The Careful Finishing of Amalgam Fillings

**Published:** 1889-04

**Authors:** Geo. F. Cheney

**Affiliations:** St. Johnsbury, Vt.


ARTICLE V.

### THE CAREFUL FINISHING OF AMALGAM FILLINGS.

BY GEO. F. CHENEY, D. D. S., ST. JOHNSBURY, VT.

In the January number of the *Archives of Dentistry*, Dr. Harrison of Cadiz, Ohio, reports a case of a molar tooth extracted by him, that had been filled on its posterior surface with amalgam, where the amalgam had been forced up over the cervical wall, between it and the wisdom tooth.

This filling became such an irritant to the surrounding tissues that absorption advanced rapidly, and when operated upon by him the wisdom tooth was destroyed, all the process gone, and a portion of the palate bone considerably absorbed, as well as much injury done to the adjacent first molar.

This case, of course, was mere carelessness, and calls to mind a similar case in my own practice, although not so extensive.

A lady came in asking for an examination of a tooth that was troubling her. Examination revealed pus oozing out from between an upper first and second molar, and by probing it was found that a mass of amalgam had been crowded between the teeth which was very difficult to remove, and upon removal proved to be nearly as large as the half of a silver three cent piece. I cannot account for room for so much to be crowded up there unless a pocket had been previously formed by food, the teeth being quite close.

It is time that some of these cases should be brought up to warn us against such unpardonable carelessness. In nearly every mouth we examine, with any number of amalgam fillings, we see evidences of carelessness in finishing, the gum instead of being healthy is of a bluish color and bleeds upon the slightest touch, and upon examination we very often find the filling over-lapping the cervical edge a thirty-second of an inch and sometimes more. The filling itself, in such cases, is enough of an irritant to keep the gum constantly inflamed, it also leaves a shoulder upon which filth is accumulated.

Too much care cannot be given the finishing of amalgam fillings. I have for the past year or so adjusted the rubber dam for most approximal fillings of amalgam, and I find enough better results can be obtained to justify the extra labor.

In most cases the dam can be adjusted without ligatures by cutting small holes and soaping the rubber on the under side and putting over four or five teeth, this makes the adjustment of the dam comparatively easy.

I work the amalgam into the cavity with bibulous paper, following Dr. Bonwill's instructions, then finish with burnishers as much as possible, and with a piece of floss silk go up between the teeth, forcing the surplus amalgam up against the dam. Clear this surplus out as much as possible, and taking off the dam will remove the remainder.

I then dismiss the patient asking him to come in for another sitting. I finish such fillings with sand-paper disks and strips.

When I cannot make this second engagement I find, by the use of the floss silk between the teeth, these fillings can be very nicely finished without leaving an over lapping mass at the cervical edge or between the teeth.

I have also found a nicely adjusted matrix of very great service and almost indispensable in some cases.

I am convinced that many of the so-called cases of ptyalism are caused by carelessness of the operator and not by the amalgam.

Furthermore, I am satisfied that a carefully inserted and a nicely finished amalgam filling, will preserve teeth as well, and cause as little inflammation of the surrounding tissues, as any other.—*Ohio Journal of Dental Science.*

